# Highly-Sensitive Textile Pressure Sensors Enabled by Suspended-Type All Carbon Nanotube Fiber Transistor Architecture

**DOI:** 10.3390/mi11121103

**Published:** 2020-12-14

**Authors:** Jae Sang Heo, Keon Woo Lee, Jun Ho Lee, Seung Beom Shin, Jeong Wan Jo, Yong Hoon Kim, Myung Gil Kim, Sung Kyu Park

**Affiliations:** 1 School of Advanced Materials Science and Engineering, Sungkyunkwan University, Suwon 16419, Korea; heojs38@gmail.com (J.S.H.); yhkim76@skku.edu (Y.H.K.); 2 Department of Electrical and Electronics Engineering, Chung-Ang University, Seoul 06974, Korea; lkw9941@gmail.com (K.W.L.); junofet@gmail.com (J.H.L.); kyocu6007@naver.com (S.B.S.); 3 Department of Electrical Engineering, University of Cambridge, Cambridge CB2 1TN, UK; jj531@cam.ac.uk; 4 SKKU Advanced Institute of Nanotechnology (SAINT), Sungkyunkwan University, Suwon 16419, Korea

**Keywords:** fiber transistors, pressure sensors, e-textile, wearable devices, active-matrix sensors

## Abstract

Among various wearable health-monitoring electronics, electronic textiles (e-textiles) have been considered as an appropriate alternative for a convenient self-diagnosis approach. However, for the realization of the wearable e-textiles capable of detecting subtle human physiological signals, the low-sensing performances still remain as a challenge. In this study, a fiber transistor-type ultra-sensitive pressure sensor (FTPS) with a new architecture that is thread-like suspended dry-spun carbon nanotube (CNT) fiber source (S)/drain (D) electrodes is proposed as the first proof of concept for the detection of very low-pressure stimuli. As a result, the pressure sensor shows an ultra-high sensitivity of ~3050 Pa^−1^ and a response/recovery time of 258/114 ms in the very low-pressure range of <300 Pa as the fiber transistor was operated in the linear region (*V*_DS_ = −0.1 V). Also, it was observed that the pressure-sensing characteristics are highly dependent on the contact pressure between the top CNT fiber S/D electrodes and the single-walled carbon nanotubes (SWCNTs) channel layer due to the air-gap made by the suspended S/D electrode fibers on the channel layers of fiber transistors. Furthermore, due to their remarkable sensitivity in the low-pressure range, an acoustic wave that has a very tiny pressure could be detected using the FTPS.

## 1. Introduction

Recently, mobile health (m-health) systems that monitor potential health issues without expert’s oversight have attracted an increasing interest [1,2,3,4]. Wearable sensor devices are essential to m-health systems because they have potential for self-diagnosis and early detection of abnormal health conditions. It is desirable for the wearable sensor devices to be integrated into apparels or attachable to human body/skin for continuous and unobtrusive monitoring of human physiology [4,5]. However, existing commercial wearable devices such as glasses, belts, watches, and wristbands are still limited in terms of social acceptance and comfortability. Thus, it is difficult to utilize the wearable devices that are used in the existing commercial devices for monitoring human physiologic signals seamlessly [1,6].

Electronic textiles (e-textiles) are the spotlighted candidates for wearable sensor devices capable of detecting various health-related physiologic signals and human activity due to their inherent mechanical properties such as flexibility, stretchabillity, wearability, breathability, and comfort to the user. An approach to embedding sensing elements in textiles is suitable for long-term monitoring with comfort and allows the freedom of sensor device’s configuration depending on the different usage [7,8,9,10,11,12,13,14]. Also, the e-textiles are compatible with traditional textile manufacturing processes such as reel-to-reel and weaving processes, resulting in scalable and cost-effective producing wearable sensor devices [15,16,17,18]. To date, for the feasibility of the textile-based wearable sensors, many research group have developed various sensor devices on textile-based substrates such as strain, pressure, and bio-physiological sensing devices using two different featured configurations: (i) Passive-type devices such as capacitive- and piezoresistive-type sensors [19,20,21,22,23,24,25] and (ii) active-type devices such as transistors [26,27,28,29,30,31]. For example, Z.L. Wang et al. reported that large-area all-textile-based pressure-sensor arrays are realized on common fabric substrates and are capable of recognizing finger movement, hand gestures, acoustic vibrations, and real-time pulse wave [32]. Also, a low-cost electronic fabric with stretchable sensor arrays can simultaneously map and quantify the mechanical stresses induced by normal pressure, lateral strain, and flexion has been explored [33]. However, even though the fabric-based sensors and arrays have a great potential in wearable sensor devices, it has mostly focused on the passive-type devices that can severely lead to signal crosstalk and high power consumption causing a restriction to develop wearable sensor systems with stable and reliable sensing performances. On the other hand, the active-type devices can address the issues mentioned above because they can allow high spatial contrast, high uniformity of array, a facile circuit integration, and a faster response [34,35]. Therefore, to realize wearable active-matrix e-textiles that retain the inherent advantages of fabric and electronic functionality, we believe that thread-like 1D fiber-based transistors are needed to be developed [36].

Herein, we demonstrate a fiber transistor-type ultra-sensitive pressure sensor (FTPS) using single-walled carbon nanotubes (SWCNTs) and thread-like suspended dry-spun carbon nanotube (CNT) fibers as an active channel layer and source (S)/drain (D) electrodes, respectively. It was found that the sensing properties of the pressure sensor are highly dependent on the contact pressure of the suspended S/D fiber electrodes. The pressure sensor showed an ultra-high sensitivity of ~3050 Pa^−1^ and an adequate response/recovery time of 258/114 ms in the very low-pressure range of <300 Pa when the fiber transistor was operated in the linear region (*V*_DS_ = −0.1 V). Due to their outstanding sensing performances, the FTPS is able to detect the sound waves from a speaker. Consequently, this new unconventional approach enables us to have access to strategic management for desirable design and implementation of the wearable e-textile health-monitoring sensors for human physiologic signal detection applications.

## 2. Materials and Methods

### 2.1. Preparation of Semiconducting Single-Walled Carbon Nanotubes (SWCNT) Solution

Semiconducting SWCNT solution was prepared by mixing High-Pressure CO (HiPCO) SWCNTs (5 mg) and poly(3-doedcylthiophene) (P3DDT; 6.25 mg) in toluene (25 mL). The solution was ultra-sonicated (BRANSON Ultrasonics Corporation, Ultrasonic processors, 400 W, Branson Sonifier 450) for 30 min at 50 °C in a temperature-controlled cooling bath. Then, the SWCNTs solution was centrifuged for 1 h at 10,000 rpm to remove SWCNT bundles and insoluble metallic SWCNT. Afterward, the supernatant in the centrifuge tube was placed into a glass vial to obtain a sorted semiconducting SWCNT solution.

### 2.2. Film Deposition and Device Fabrication

For the bottom gate and top thread-like S/D contact of a fiber transistor, Cr (88 nm) was deposited on an optical fiber substrate by using an RF-magnetron sputtering system. Then, Al_2_O_3_ gate dielectric layer was formed on the Cr-gate electrode by using an atomic-layer-deposition (ALD) system at 150 °C. Subsequently, in order to form SWCNT network as a channel layer on the ALD-deposited Al_2_O_3_ film, the SWCNTs solution was coated by using a customized dip-coater with a withdrawal speed of 0.78 mm·s^−1^. Finally, for the S/D contact electrode, the conductive dry-spun CNT fibers with a diameter of about 83.3 µm (~1683.6 S/cm) were placed on a TFT’s body composing of Cr-gate, ALD-Al_2_O_3_ gate dielectric, and SWCNT channel layers by using a sewing process. Here, note that the sewing process is utilized to embed the TFT’s body in a textile substrate and to design the suspended S/D electrode configuration. In addition, in the case of the fabrication of the fiber transistor-type pressure sensors, the TFT’s body was embedded in the textile substrate and then the CNT fiber S/D electrodes were suspended on the TFT’s body to form the air-gap between the S/D and channel layers. Here, a commercial woven fabric composed of 55% linen and 45% cotton was used as a textile substrate.

### 2.3. Device Characterizations

For the surface morphology of CNT fibers, a field-emission scanning electron microscopy (FESEM) (ZEISS Microscopy, Supra40VP, Carl Zeiss) was performed. In addition, in order to achieve the electrical characteristics of the CNT fibers, two ends of the fiber were connected with a silver paste and then the electrical resistance was measured by a source meter (Keithley, Model 2450 Source-meter Instrument). Note that the conductivity was extracted by using the cross-section area (*A* = 5.44 × 10^−5^ cm^2^, the surface area) of the cylindrical-shaped CNT fibers.

The electrical characteristics of the ALD-deposited Al_2_O_3_ such as the C-F and J-E curves were extracted from a metal/insulator/metal structures (MIM; Cr/Al_2_O_3_/Au) with an LCR meter (Agilent Technologies, Inc., Santa Clara, CA, USA, Agilent 4284A) and a semiconductor parameter analyzer (Agilent Technologies, Inc., Agilent 4156C). The fabricated fiber SWCNT transistors and the relative change in current of the FTPS under pressure were analyzed by the semiconductor parameter analyzer in air condition at room temperature. For the dynamic measurement of the sensor, a measuring system consisting of a dual channel source meter (Keithley, Model 2636B Source-meter Instrument) and a data acquisition system were utilized. Pressure forces were performed with a motorized test stand (Mark-10, ESM 303). Also, for the detection of an acoustic wave, a speaker was located on the sensor device away from approximately 2–3 cm and turned on and off repeatedly.

## 3. Results and Discussions

**Figure 1:**
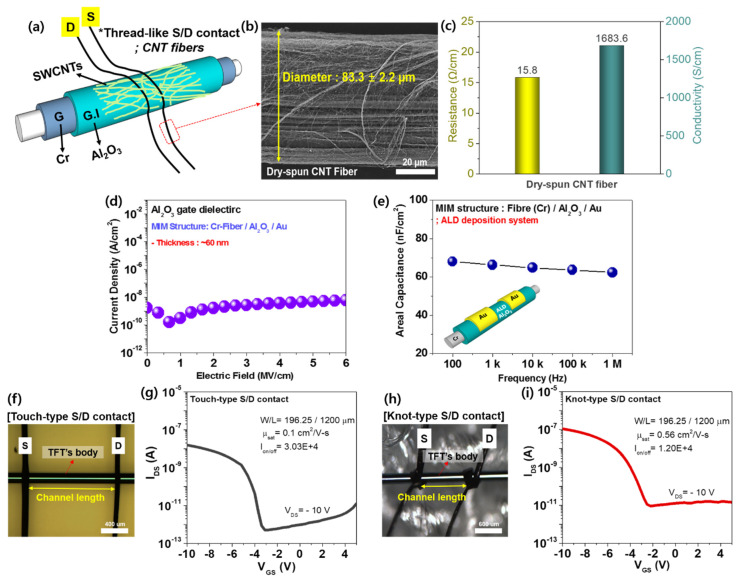
(**a**) A schematic of an ultra-sensitive fiber transistor-type pressure sensor (FTPS) with thread-like carbon nanotube (CNT) fiber S/D electrodes. (**b**) A field-emission scanning electron microscopy (FESEM) image and (**c**) the electrical conductivity/resistance of the dry-spun CNT fiber. The electrical characteristics of atomic layer deposition (ALD)-deposited Al_2_O_3_ gate dielectric on a 1-D fiber substrate; (**d**) the leakage current density versus electric-field (J-E) and (**e**) the areal capacitance versus frequency (C-F) curves. The optical images that show the channel length (1200 µm) and the thin-film transistor’s (TFT’s) body consisting of three components such as gate, gate dielectric, and channel layers, and the transfer characteristics of the FTPS with (**f**,**g**) the touch-type S/D contact and (**h**,**i**) the knot-type S/D contact.

In general, the previous transistor-type pressure sensors used an air-gap dielectric layer between a channel layer and a top suspended gate electrode as a pressure sensing element [37,38,39,40]. However, despite their enhanced sensing performances, the processing-cost and complex manufacturing process still remain as a challenge to be addressed. In this study, in order to address the issues, we demonstrate an ultra-sensitive fiber transistor-based pressure sensor (FTPS) using a simple approach that utilized thread-like dry-spun CNT fiber source (S)/drain (D) electrodes, as shown in Figure 1a. The FTPS is mainly composed of both the thin-film transistors (TFT) body including an optical fiber, gate (G), and gate dielectric (G.I) and the thread-like dry-spun CNT fibers for the S/D electrode. For the formation of the TFT’s body, Cr gate electrode and Al_2_O_3_ gate dielectric were deposited on the optical fiber substrate by using a radio-frequency magnetron sputtering and atomic layer deposition (ALD) system, respectively. Then, semiconducting SWCNT network was formed on the ALD-deposited Al_2_O_3_ as a channel layer. Finally, for the S/D electrodes, two thread-like dry-spun CNT fibers with a distance of 1200 µm (channel length) were placed on the SWCNT channel layer. As shown in Figure 1b, the dry-spun CNT fiber exhibited a diameter of 83.3 ± 2.2 µm and a micro-network structure. In addition, they showed the electrical conductivity and resistance of 1683.6 S/cm and 15.8 Ω/cm, respectively, which indicates that the dry-spun CNT fibers can be used as the S/D electrodes for SWCNT transistors (Figure 1c). However, even though the optical fiber substrates used in this study showed their own mechanical flexibility due to a small size diameter (~200 µm), it might be damaged under extreme deformation conditions, which should be addressed for the future work. Figure 1d,e show the leakage current density versus electric field (J-E) and the areal capacitance versus frequency (C-F) characteristics of the Al_2_O_3_ gate dielectric layer on the fiber substrate, respectively, which are extracted by a metal-insulator-metal (MIM; Cr/Al_2_O_3_/Au) configuration. The 1-dimensional (1-D) ALD-deposited Al_2_O_3_ gate dielectric layer with a thickness of 60 nm exhibited the low leakage current density of 5.19 × 10^−8^ A cm^−2^ at 3 MV cm^−1^ and the areal capacitance of 66.2 nF cm^−2^ at 1 kHz, which means that the Al_2_O_3_ gate dielectric with good insulating properties might contribute to the stable operation of fiber-based transistors.

Moreover, to confirm that our fiber-based SWCNT transistor with thread-like CNT fiber S/D electrodes is appropriately operated, we firstly investigate the electrical characteristics of the fiber transistor with two different S/D electrode contact-mode types such as touch-type and knot-type. Figure 1f,h show the optical image and transfer curve of the fiber-transistors with touch-type and knot-type S/D contact, respectively. Note that the channel length was defined as a distance between two CNT fiber electrodes as shown in the optical images. We observed that the thread-like S/D contacts are well-formed on the TFT’s body and two samples exhibited p-type behavior with saturation mobility of 0.1 cm^2^ V^−1^s^−1^ and 0.56 cm^2^ V^−1^s^−1^ for the touch-type and knot-type S/D contact, respectively (Figure 1g,i). In addition, from the results, it was found that the electrical performances of the fiber transistors rely on the contact pressure of the CNT fiber S/D electrodes. As a result, it is expected that the fiber transistor can be used as a pressure sensor device according to the contact configuration of the CNT fiber S/D electrodes.

**Figure 2:**
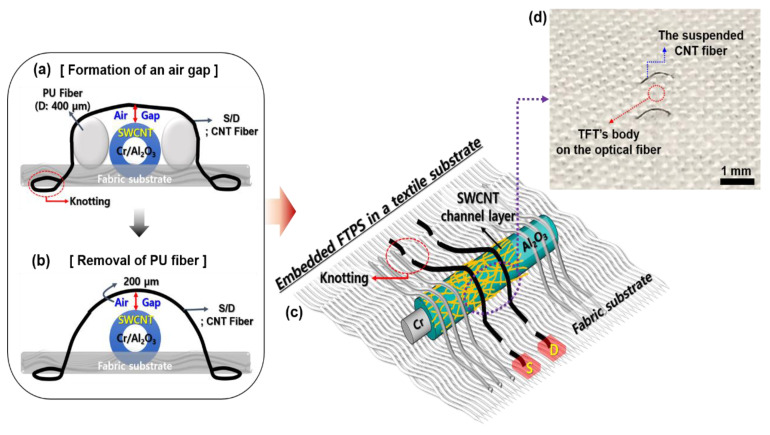
Schematic of the fabrication process the fiber transistor-type pressure sensors (FTPS); (**a**) Formation of an air gap with sacrificial PU fibers and a sewing process, and (**b**) the FTPS structure after removing of the PU fibers. (**c**) Schematic illustration of the embedded FTPS in a textile substrate with the suspended CNT fibers. (**d**) A photograph of the practical FTPS showing the TFT’s body and the suspended CNT fiber.

To fabricate an ultra-sensitive fiber transistor-type pressure sensor (FTPS), the fiber-based SWCNT TFT’s body including the optical fiber, gate (Cr), and gate dielectric (ALD deposited Al_2_O_3_) was embedded in textiles and subsequently the conductive CNT fibers with the resistance of 15.8 Ω cm^−1^ and diameter of ~83.3 µm were suspended as a S/D electrode on the TFT’s body by using a sewing process. Note that the CNT fibers were tightly knotted to maintain the suspend S/D electrode structure on the fabric substrate. In addition, Figure 2a shows that sacrificial polyurethane (PU, a diameter of ~400 µm) fibers were used to provide an air gap between the suspended S/D electrodes and the SWCNT channel layer, resulting in the air gap distance of approximately 200 µm. Then, after removing the sacrificial PU fibers, the air gap could be preserved by the intrinsic elasticity of conductive CNT-based fibers used as the suspended S/D electrode, as shown in Figure 2b [41,42]. Finally, the FTPS device’s configuration not only could maintain the air gap but also preserve it during a repetitive cyclic loading/unloading. Figure 2c,d show the embedded FTPS in a textile substrate with the suspended CNT fibers and the practical fabricated FTPS device, respectively, showing the air gap between the TFT’s body and the suspended CNT fiber.

**Figure 3:**
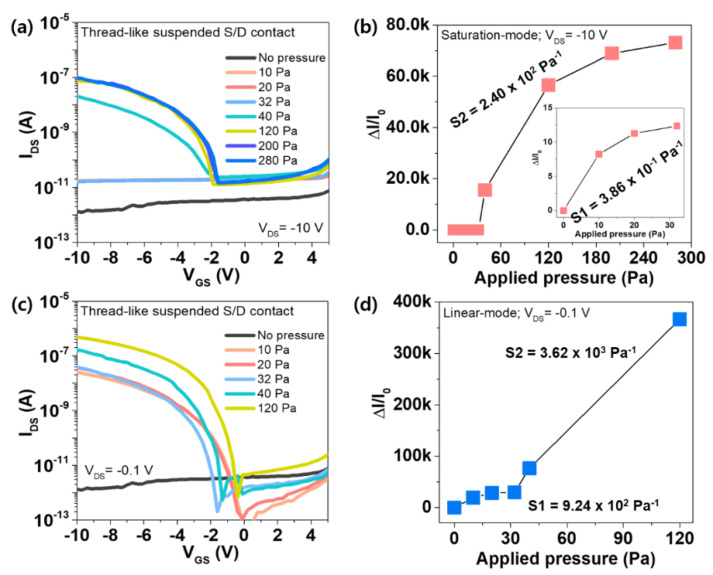
(**a**) The transfer curve of the FTPS device under different applied pressures at *V*_DS_ = −10 V (saturation-mode). (**b**) The relative change in the drain-source current (*I*_DS_) of the FTPS as a function of the applied pressure from 0 to 280 Pa in the saturation-mode. (**c**) The transfer curve of the FTPS device under different applied pressures at *V*_DS_ = −0.1 V (linear-mode). (**d**) The relative change in the drain-source current (*I*_DS_) of the FTPS as a function of the applied pressure from 0 to 120 Pa in the linear-mode.

We also measured the pressure dependent performance of the FTPS to investigate the sensing characteristics of the fabricated pressure sensor. Figure 3 presents the transfer characteristics and the corresponding relative changes in the drain current (Δ*I*/*I*_0_), where I_0_ is the initial current with no pressure and Δ*I* = *I* − *I*_0_, of the FTPS in the saturation and linear regions under different applied pressures ranging from 0 to 280 Pa. In both cases, the drain-source current (*I*_DS_) increased with increasing the applied pressure at *V*_DS_ = −10 V and −0.1 V and a low-limit of detection was 10 Pa, indicating that the fiber-based SWCNT transistor with thread-like suspended S/D electrodes can response to very low pressure stimuli (Figure 3a,c). Interestingly, there is a significant difference in the sensitivity of the FTPS between two different regions as shown in Figure 3b,d. In the case of the saturation region (*V*_DS_ = −10 V), the FTPS showed the sensitivity of 3.86 × 10^−1^ Pa^−1^ and 2.4 × 10^2^ Pa^−1^ in the pressure range of 0~32 Pa and 32~280 Pa, respectively. On the other hand, with the linear region (*V*_DS_ = −0.1 V), it was observed that the sensitivity is considerably enhanced in the pressure range of 0~120 Pa. Despite higher sensitivity, however, the sensing range was degraded from 120 Pa to 280 Pa compared to the saturation-mode. In particular, in the low pressure range (<~40 Pa), linearity was more improved than a high V_DS_ as well as sensitivity. The result might be attributed to that the transistor is operated entirely in the subthreshold region, resulting in low-off current levels and exponentially increased channel current with the gate bias [43]. Thus, the low V_DS_ (linear-mode) would induce linearly higher current changes upon pressure. At the high V_DS_ (saturation-mode), the operation of the transistors would be shifted to out of the subthreshold region, showing high off-current and slow current change with applying pressure. The results indicate that the ultra-high sensitive FTPS device to low pressures not only enables the potential applications that require high sensitivity in the low-pressure but also their pressure-sensing properties can be controlled by the operating modes of the transistors, expanding the possibilities of the FTPS in wearable healthcare applications. For comparison with previously reported literatures, the comparison of the electrical performances of our sensors with existing pressure sensors is listed in Table 1.

**Figure 4:**
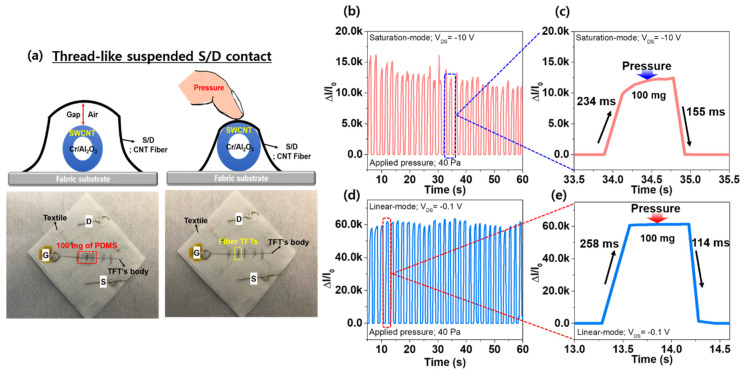
(**a**) The mechanical and electrical mechanism of the embedded FTPS with the thread-like CNT fiber S/D electrodes in a textile substrate under pressure. The dynamic response characteristics of the sensor under the applied pressures of 40 Pa and the response/recovery times during the cyclic pressure loading-unloading test at the saturation-mode (**b**,**c**) and the linear-mode (**d**,**e**).

In addition, to evaluate the real-time stable operating pressure sensing properties, we performed the cyclic pressure loading-unloading tests with the FTPS (applied pressure = 40 Pa). As aforementioned above, for the dynamic current response to applying the pressure of 40 Pa, the motorized test stand measuring a gauge force was implemented. Additionally, a piece of PDMS with a weight of 100 mg that is about 40 Pa was placed on the FTPS with thread-like suspended S/D electrodes embedded in the textile, as shown in Figure 4a. As a result, the FTPSs operated in both the saturation and linear modes exhibited a stable pressure response characteristic with the average response and recovery time of ~246 ms and ~134 ms, respectively, indicating that this result may enable real-time monitoring against subtle pressures such as human physiological signals with exceptional sensitivity and good stability (Figure 4b–e). Notably, the FTPS’s pressure sensing characteristics are attributed to the thread-like CNT fiber S/D electrodes suspended in the textile substrate. With the suspended S/D electrode’s configuration, applying pressure would cause a decrease of the air gap between the top CNT fiber S/D electrode and the SWCNT channel layer and then a contact pressure occurred with the absence of the air gap, simultaneously. Such contact pressure will be increased and induce an intimate contact between the S/D and channel regions with increasing applied pressure, resulting in an increase of the I_DS_ in the fiber transistors (Figure 4a). However, as shown in Figure 4b,d, there are some amplitude fluctuations under the pressure of 40 Pa. The results might be caused by the fringe effect originated from unpatterned semiconductor active layers and gate electrodes. In general, the fringe effect can negatively influence the electrical characteristics, showing parasitic capacitance, gate leakage current, and off-current increased [44,45]. As a result, the corresponding amplitude under the dynamic response test might be slightly fluctuated by the undesirable operation of the FTPS devices originated from the fringe effect. Therefore, the patterning process for the SWCNT channel and the Cr gate electrode is encouraged to obtain desirable output signals in transistor-type pressure sensor applications.

**Figure 5:**
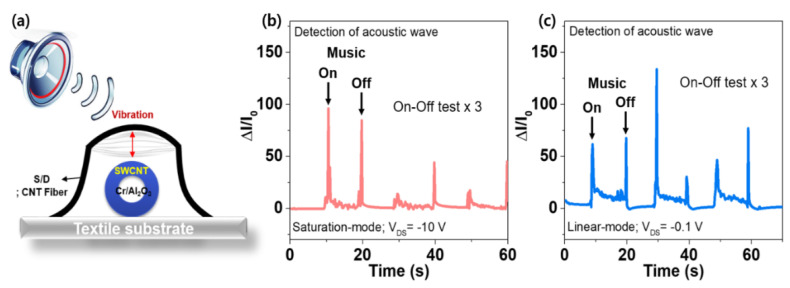
(**a**) Schematic illustration of sensor device’s configuration for the detection of an acoustic wave. The relative changes in the I_DS_ of the FTPS while the music sound repeatedly turned on and off at (**b**) *V*_DS_ = −10 V and (**c**) −0.1 V.

As a feasible demonstration of a fiber transistor-type sensor embedded in textiles for the real-time detection of acoustic waves, the applicability of the FTPS devices was performed by applying music sound, as shown in Figure 5a. For the response characteristics to an acoustic wave, the Δ*I*/*I*_0_ of the FTPS devices were measured while the music sound repeatedly turned on and off. Figure 5b,c show that the FTPS devices can detect a very tiny acoustic wave in the two different modes as well as the operation of a speaker, which is because the contact pressure between the S/D and channel layers would be continuously generated by the vibration made by the acoustic wave during the music sound turned on. Despite high sensitivity in the range of the low-pressure, the FTPS devices are still not enough to be applied to practical applications due to non-optimization of a new device’s configuration as well as the properties for the suspended S/D electrodes. However, compared to conventional two-terminal sensors such as resistive- and capacitance-types that have only one-sensing parameter, the transistor-type pressure sensor is more suitable for practical applications because they have various electrical parameters such as threshold voltage, subthreshold swing, and off-current, etc. Accordingly, we believe that with this methodology and fine-tuning the properties of the suspended S/D electrodes, it may be possible to construct sensor devices capable of continuously monitoring heart sounds or other body sounds in the future. Furthermore, it may be enabled to develop cost-efficiently ultra-high sensitive e-textile based pressure sensors using a simple textile-manufacturing process in the future.

## 4. Conclusions

We demonstrate an ultra-sensitive pressure sensor capable of detecting a very low-pressure using a new architecture of a fiber SWCNT transistor. In particular, for the high-sensitive fiber transistor-type pressure sensors (FTPS), the conductive CNT fibers were used as the S/D electrodes and placed on the TFT’s body consisting of Cr-gate, Al_2_O_3_ gate dielectric, and SWCNT channel layers on a 1-D fiber substrate. As a result, the pressure-sensing characteristics of the FTPS are dependent on the existence and nonexistence of the contact pressure between the top CNT fiber S/D electrode and the SWCNT channel layer. Additionally, it can be controlled by the operating mode of the fiber transistors (saturation and linear modes). The FTPS devices exhibited an ultra-high sensitivity of ~3050 Pa^−1^ in the very low-pressure range of <300 Pa, showing that the pressure sensors may enable developing wearable healthcare applications that require high sensitivity in the low-pressure. Moreover, an acoustic wave could be detected using the FTPS with thread-like CNT fiber S/D electrodes suspended in a textile substrate due to its excellent high sensitivity. We believe that a new fabrication approach of a fiber transistor-type pressure sensor will advance potential applications of e-textile wearable electronics for the detection of human physiologic signals.

## Figures and Tables

**Table 1:** Comparison of the sensing performances of the fiber and transistor-type pressure sensors.

Material/Structure	Sensitivity	Sensing Range	Limit of Detection	Ref.
CNT-coated Fabric and VHB	14.4 kPa^−1^	0–15 kPa	2 Pa	[32]
Woven structure				
AgNW and C-PDMS coated fibers	4.29 N^−1^	0–20 N	0.03 N	[33]
Coaxial structure				
SBS polymer and Ag nanoparticle fibers	0.21 kPa^−1^	0–10 kPa	0.05 N	[36]
Perpendicular cross structure				
Air-dielectric IGZO FETs with G-PDMS	1.1 × 10^−3^ kPa^−1^	200–5 MPa	0.21 kPa	[38]
(Transistor-type)				
Organic TFTs with suspended gate	192 kPa^−1^	100–5 kPa	0.5 Pa	[39]
(Transistor-type)				
Graphene FETs with air-dielectric	2.05 × 10^−4^ kPa^−1^	250–3 MPa	5 Kpa	[40]
(Transistor-type)				
